# Exploring the Association Between Patient–Nurse Ratio and Nurses' Occupational Stressors: A Cross-Sectional Study

**DOI:** 10.1155/jonm/6160674

**Published:** 2025-05-24

**Authors:** Yi-Chuan Chen, Hsueh-Ching Wu, Jiune-Jye Ho, Nai-Yun Cheng, Yue Leon Guo, Judith Shu-Chu Shiao

**Affiliations:** ^1^School of Nursing, College of Medicine, National Taiwan University (NTU), Taipei, Taiwan; ^2^Department of Nursing, National Taiwan University Hospital, Taipei, Taiwan; ^3^Department of Nursing, Hsin Sheng Junior College of Medical Care and Management, Taoyuan City, Taiwan; ^4^Center for Occupational Accident Prevention and Rehabilitation (COAPRE), New Taipei City, Taiwan; ^5^Institute of Labor, Occupational Safety and Health (ILOSH), Ministry of Labor, New Taipei City, Taiwan; ^6^Environment and Occupational Medicine, College of Medicine, National Taiwan University (NTU) and NTU Hospital, Taipei, Taiwan; ^7^Graduate Institute of Environmental and Occupational Health Sciences, College of Public Health, National Taiwan University (NTU), Taipei, Taiwan; ^8^Susan Wakil School of Nursing and Midwifery, The University of Sydney, Sydney, New South Wales, Australia

**Keywords:** nurses, occupational stress, spline, staffing, working conditions, workplace

## Abstract

**Background:** The patient-nurse ratio significantly influences nursing workloads, but its specific relationship with nurses' occupational stressors is poorly understood.

**Aim:** This study aimed to examine the association between patient-nurse ratio and occupational stressors among nurses, highlighting understaffing as a potential driver of stress in clinical environments.

**Methods:** A self-administered questionnaire was distributed to full-time nurses in the medical and surgical wards of accredited hospitals. Data collected included the average daily patient-nurse ratio, subscale scores from the Nurses' Occupational Stressor Scale (NOSS), and demographic and workplace variables such as sex, age, educational attainment, marital status, hospital ownership, unit type, major shift in the past 3 months, work tenure, sleeping hours, and weekly working hours. Logistic regression models and restricted cubic splines were used to analyze associations between the average daily patient-nurse ratio and elevated nursing stressors. The study followed the STROBE guidelines for cross-sectional research.

**Results:** Among the 996 nurses surveyed, a higher average daily patient-nurse ratio was significantly associated with increased stress levels across all subscales of the NOSS. Restricted cubic spline analysis revealed that a lower average daily patient-nurse ratio corresponded to reduced probabilities of encountering higher stressors related to work demands, insufficient support from coworkers or caregivers, organizational challenges, and difficulty taking leave. Conversely, higher average daily patient-nurse ratios were linked to greater stress probabilities in all measured domains.

**Conclusion:** This study demonstrates that higher average daily patient-nurse ratios significantly increase occupational stress among nurses. Reducing the patient-nurse ratio may mitigate these stressors and improve the overall well-being of nursing staff.

## 1. Introduction

Achieving universal health coverage (UHC) requires a strong and adequately staffed workforce, especially nurses, who make up the largest segment [1]. The World Health Organization (WHO) has emphasized the urgency of strengthening this workforce and identified safe staffing—particularly manageable patient-to-nurse ratios (PNRs)—as a global policy priority [2, 3].

Despite these efforts, nursing understaffing remains prevalent and predates the COVID-19 pandemic. It is linked to increased intention to leave [4], higher turnover [5], excessive workloads [6], greater burnout and job dissatisfaction [4, 7], poor cardiovascular health [8], and compromised patient safety [9]. In response, several governments, such as the United States, Japan, Taiwan, Australia, and the United Kingdom, have enacted or are considering PNR legislation [10–15].

Research has consistently demonstrated that improved staffing levels reduce adverse events [16], decrease hospital stays [17], and enhance hospitals' ability to attract qualified personnel and deliver better care.

In Taiwan, the development of PNR regulations has been gradual. In 2013, hospital accreditation guidelines piloted a nurse staffing ratio test. By 2017, the Ministry of Health and Welfare mandated monthly public disclosure of nurse staffing ratios. In 2019, the all-day average PNR was officially included in the Establishment Standards for Medical Institutions [12]. Most recently, in 2024, the Ministry introduced a three-shift PNR policy, offering incentives to hospitals that comply. Despite these steps, many institutions continue to struggle with meeting the required ratios, highlighting persistent concerns about staffing adequacy [18, 19].

The scope of nursing work is extensive, encompassing direct patient care, administrative duties, and interprofessional collaboration. To assess workplace stressors, researchers have developed tools such as the Expanded Nursing Stress Scale [20], the Practice Environment Scale of the Nursing Work Index [21], and the Nurses' Occupational Stressor Scale (NOSS) [22]. Some stressors stem directly from patient care [23, 24], while others arise from inadequate staffing, organizational culture, or team dynamics. Although the association between nurse stress and staffing shortages is well-documented [5, 7, 23], the specific connection between PNRs and distinct occupational stressors remains underexplored.

Despite ongoing healthcare reforms, further empirical research is needed to clarify how PNR influences specific stressor domains, rather than viewing nurse stress as a singular outcome. Most existing studies examine aggregated stress measures, without pinpointing which occupational stressors are most sensitive to staffing levels [25]. Clarifying these distinctions is essential for designing targeted strategies to support nurse well-being and retention.

Building upon a nationwide survey that examined multiple aspects of nurses' work environments, this study focuses specifically on the association between PNRs and occupational stressors. Previous analyses using this dataset identified significant links between poor psychological work environments and suicidal ideation [26], and between high standardized PNRs and increased burnout, job dissatisfaction, and intention to leave [4]. The present study extends this body of research by exploring how PNRs relate to distinct domains of occupational stressors using a validated multidimensional stress scale.

Therefore, this study aimed to investigate the association between PNRs and nurses' occupational stressors using the NOSS. Specifically, we sought to identify which stressor domains were most affected by varying PNR levels and whether a nonlinear relationship existed. These findings are intended to inform staffing policies and workplace interventions that support nurse well-being and workforce sustainability.

## 2. Methods

### 2.1. Research Design

This cross-sectional study used a self-administered questionnaire conducted in Taiwan in 2014, adhering to the STROBE reporting guidelines.

### 2.2. Participants

To ensure regional representativeness, hospitals were categorized into four major regions: Northern, Central, Southern, and Eastern/offshore islands. Stratified random sampling was applied, with one hospital randomly selected per region, followed by every 10th hospital from a list of accredited hospitals that met the conformity and excellence criteria under the New Hospital Accreditation program by the Ministry of Health and Welfare (2010–2013).

A total of 71 hospitals were contacted, and 57 agreed to participate. The sample included tertiary, secondary, and primary hospitals, covering institutions of varying sizes and staffing levels.

Anonymous self-administered questionnaires were distributed to 2%–5% of registered nurses at each hospital, yielding 3974 distributed questionnaires and 3786 returns. Responses from nurse managers or nurse practitioners (*n* = 237) and incomplete submissions for the NOSS (*n* = 323) were excluded, resulting in a response rate of 81.2%.

The required sample size was calculated using G^∗^Power 3.1.9.7. Assuming a small effect size (*f*^2^ = 0.02) [27], significance level (*α* = 0.05), power of 0.8, and 13 predictors, the minimum required sample size was 904.

**Figure 1:**
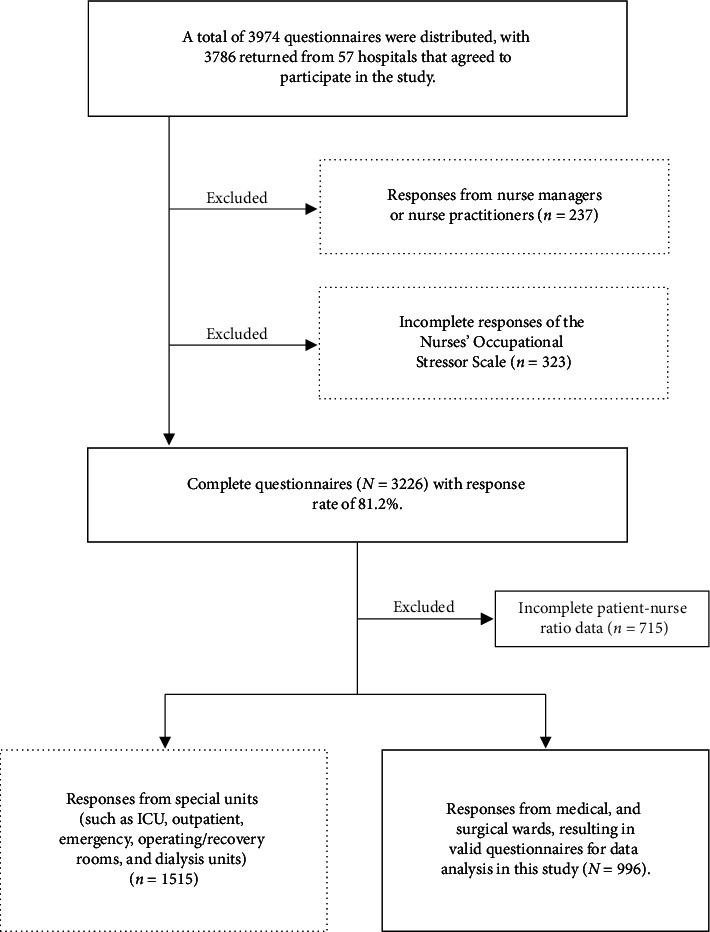
Flowchart of data selection.

Further data exclusions were applied based on the Establishment Standards for Medical Institutions [12], which define PNR requirements for acute general hospital beds. Respondents with incomplete PNR data (*n* = 715) were removed. To ensure comparability with general acute care PNR frameworks, nurses working in specialized units (e.g., ICU, outpatient, emergency, operating/recovery rooms, and dialysis units; *n* = 1515) were also excluded because of distinct staffing structures in these areas. Ultimately, 996 valid questionnaires were included in the final analysis, exceeding the required sample size.  Figure 1 illustrates the data selection process.

### 2.3. Data Collection and Measures

Data collection was conducted from August to December 2014. Paper-based, anonymous, self-administered questionnaires were distributed and collected by designated nursing administrators at each participating hospital. Each nurse was allowed to complete the questionnaire only once, and no identifying information (e.g., names or employee IDs) was collected to ensure confidentiality and minimize response bias. A small token gift valued at approximately USD 2 was provided to encourage participation. Measures were taken to prevent duplicate submissions, such as the use of controlled distribution lists and collection procedures supervised by local coordinators.

#### 2.3.1. Patient-Nurse Ratio

Hospitals in Taiwan are classified into three levels based on their healthcare facilities, providers, and services: primary (district hospitals), secondary (regional hospitals), and tertiary (medical centers). Primary hospitals provide general-level emergency care, secondary hospitals offer moderate-level care, and tertiary hospitals deliver advanced-level care. The Taiwanese standard of the PNR is legislated as the daily average number of patients per nurse for acute general beds, which is set at 9, 12, and 15 for tertiary, secondary, and primary hospitals, respectively [12].

A previous survey [4] assessed PNR through the question: “Generally speaking, how many patients do you care for during the day, evening, and night shifts?” The average daily PNR (ADPNR) was calculated using the following formula:

(1) $$\begin{array}{c} \text{ADPNR}=\text{AVERAGE}\left(\frac{1}{\left(1/D\right)+\left(1/E\right)+\left(1/N\right)}\right), \end{array}$$

where *D*, *E*, and *N* represent the number of patients under the care of each nurse during the day, evening, and night shifts, respectively [4].

#### 2.3.2. NOSS

The NOSS was originally developed and validated in Taiwan, ensuring its applicability to the healthcare system and nursing work environment [22]. The initial version included 43 items, later condensed to 21 items to evaluate nursing-specific stressors. The scale development process involved a comprehensive literature review, focus group discussions with frontline nurses, expert feedback, and pilot evaluations, ensuring that it accurately captured occupational stressors unique to Taiwanese hospital nurses [22]. Both the original Chinese version [22] and the translated Turkish version [28] demonstrated good construct validity and test-retest reliability, supporting its robustness across different healthcare settings. In addition, the scale effectively predicted personal burnout, client-related burnout, job dissatisfaction, and intentions to leave among hospital nurses [22]. Given that nursing stressors vary across different healthcare systems, the NOSS explicitly considers key factors relevant to Taiwan's nursing practice environment, such as on-call shift frequency, reliance on family members for patient care, and financial reimbursement for extra working hours [22].

The 21-item NOSS consists of nine subscales: work demands, work-family conflict, insufficient support from coworkers or caregivers, workplace violence and bullying, organizational issues, occupational hazards, difficulty taking leave, powerlessness, and unmet basic physiological needs. Each item is rated on a four-point Likert scale (1 = *strongly disagree* to 4 = *strongly agree*), with higher scores indicating more frequent stressful events [22]. To standardize scores across the subscales, total subscale scores were adjusted to a scale of 0–100, accounting for differences in the number of items across subscales.

#### 2.3.3. Potential Confounders

Several potential confounders were considered to better understand the association between PNR and nurses' stressors, as identified in previous studies. Demographic factors included age [29], work tenure, educational attainment, marital status [30], and average daily sleeping hours during workdays [31]. Workplace characteristics included hospital sector (public vs. private), hospital level (primary, secondary, and tertiary), unit type, primary shift over the past 3 months, and weekly working hours [4, 31]. Potential confounders were categorized as follows: age (≤ 25, 26–35, ≥ 36 years); educational attainment (under college, college or above); marital status (single/divorced/separated/widowed, married/cohabiting); sleeping hours per workday (< 6, ≥ 6); hospital level (primary, secondary, and tertiary); hospital ownership (public, private); unit type (medical, surgical); predominant shift over the past 3 months (day, evening, night, or rotating); and weekly working hours (< 55, ≥ 55).

### 2.4. Statistical Analysis

Descriptive statistics were employed to summarize the distributions of participants' demographic characteristics, working conditions, and scores across the subscales of the NOSS. The 75th percentile score for each NOSS subscale was used as the threshold for identifying higher stress levels. Logistic regression models were utilized to evaluate the association between the ADPNR and NOSS subscales, adjusting for potential confounders [32]. Each of the nine logistic regression models included ADPNR as a consistent predictor, while other covariates were selectively incorporated based on their significance in preliminary univariate analyses.

To handle missing data, participants with incomplete NOSS responses were excluded using listwise deletion, as illustrated in Figure 1. For other individual-level variables (such as age, marital status, and educational achievement), missing values were retained and coded as missing. Outliers were not excluded, as variable ranges were reviewed and determined to be reasonable based on previous research and clinical relevance.

Data analyses were conducted using two-tailed tests in JMP Pro version 17.0 (SAS Institute, Cary, North Carolina). Statistical significance was set at a *p* value of < 0.05. Dose-response relationships were assessed using restricted cubic splines, with knots positioned at the 10th, 50th, and 90th percentiles of ADPNR. These analyses were performed using SAS version 9.4 (SAS Institute, Cary, North Carolina) [33].

### 2.5. Ethical Considerations

The study protocol was approved by the National Taiwan University Hospital Research Ethics Committee (Approval No. 201407075RINA). Participants were provided with detailed information regarding the study's objectives, potential risks and benefits, measures to ensure confidentiality, and their right to withdraw at any time. This information was included in the cover letter accompanying the questionnaire. Written consent was waived to preserve anonymity, and returning the completed questionnaire signified participants' consent to participate.

## 3. Results

### 3.1. Participants' Characteristics

The demographic and work-related characteristics of the participants are summarized in Table 1. Most participants were female (*n* = 950, 95.4%) and single (*n* = 656, 65.9%), with a mean age of 30.6 years (SD = 6.9). A majority had completed college or higher education (*n* = 662, 66.5%) and reported an average current work tenure of 6.8 years and total work tenure of 8.2 years. The distribution of participants across hospital levels was relatively even: primary hospitals (31.6%), secondary hospitals (32.7%), and tertiary hospitals (35.6%). Notably, 40.0% of the participants had predominantly worked day shifts over the past 3 months. Nurses reported an average of 6.9 h of sleep per night and 9.5 work hours per day, with an average weekly workload of 47.6 h. The ADPNRs for primary, secondary, and tertiary hospitals were 13.1, 12.6, and 10.9, respectively.

### 3.2. NOSS Subscale Distribution

The scores for the nine NOSS subscales, adjusted to a range of 0–100, are presented in Table 2. The mean scores ranged from 61.4 to 74.1, with the highest scores observed in the Work Demands subscale.

### 3.3. Association Between Characteristics and NOSS Subscales

Logistic regression models were applied using the 75th percentile score of each subscale as a cutoff to define higher stress levels. Crude odds ratios (ORs) are presented in Table 3, and mutually adjusted ORs are shown in Table 4. While univariate results revealed a consistent association between higher ADPNR and all elevated stressor domains, the adjusted models provide a more accurate estimation by accounting for covariates. Specifically, a one-unit increase in ADPNR was associated with adjusted ORs ranging from 1.07 to 1.18 across the nine stressor subscales (Table 4). The strongest associations were observed for unmet basic physiological needs (adjusted OR = 1.18, 95% CI: 1.10–1.25), work demands (adjusted OR = 1.13, 95% CI: 1.06–1.20), organizational issues (adjusted OR = 1.12, 95% CI: 1.06–1.19), and difficulty taking leave (adjusted OR = 1.11, 95% CI: 1.05–1.17). These results indicate that increases in patient load per nurse are linked to significantly heightened stress levels across diverse domains.

Other factors were also associated with stress levels. Nurses aged 26–35 reported lower perceived support from coworkers or caregivers and greater difficulty taking leave. Married or cohabitating nurses experienced more work-family conflict. Those with college or higher education qualifications expressed greater work-family conflict and stronger feelings of powerlessness.

Work tenure appeared to buffer stress, with longer tenure correlating with reduced work demands and fewer unmet basic physiological needs. However, employment in tertiary hospitals was associated with more organizational issues, higher occupational hazards, and greater unmet basic physiological needs. Similarly, nurses in surgical wards faced elevated stress from higher work demands, insufficient support from coworkers or caregivers, organizational issues, and occupational hazards.

Nurses working on rotating shifts over the past 3 months increased workplace violence and bullying as well as more organizational issues. Moreover, working ≥ 55 h per week was linked to eight of the nine subscales of the NOSS (adjusted ORs ranging from 1.54 to 2.78), excluding workplace violence and bullying.

**Figure 2:**
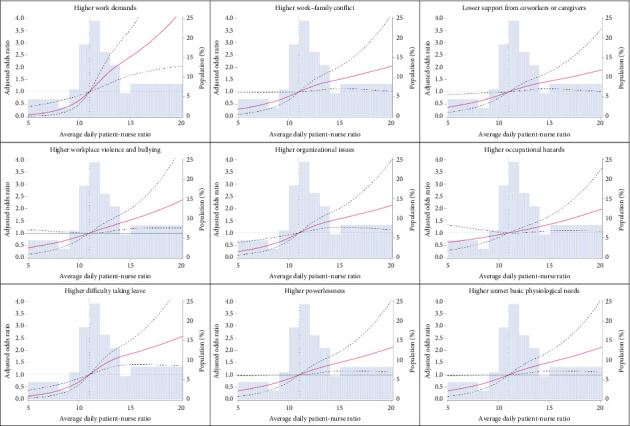
Associations between average daily patient-nurse ratio and subscales of the Nurses' Occupational Stressor Scale, using restricted cubic spline. The histogram illustrates the distribution of participants across average daily patient-nurse ratio groups. The lilac line depicts the adjusted odds ratio (OR), while the dashed line represents the 95% confidence interval. For the restricted cubic spline analysis, knots were positioned at the 10th, 50th, and 90th percentiles of the average daily patient-nurse ratio. The 25th percentile, approximately 11, of the average daily patient-nurse ratio was designated as the reference point, with the adjusted OR set to 1.0.

Figure 2 illustrates dose-response relationships between ADPNR and stressors using restricted cubic splines. The reference point (OR = 1) was set at an ADPNR of 11, approximately the 25th percentile. Lower ADPNR levels were associated with decreased probabilities of encountering higher work demands, lower levels of coworker or caregiver support, organizational issues, and greater difficulty taking leave. Conversely, an ADPNR exceeding 11 substantially increased the likelihood of experiencing all nine heightened NOSS stressors.

## 4. Discussion

This study departs from prior research that broadly categorized PNR as a nursing stressor [34, 35], offering novel insights by identifying specific stressor domains linked to varying ADPNR levels. The cubic spline analysis identified a threshold of ADPNR = 11, below which protective effects were observed in four key domains: reduced work demands, better support from coworkers and caregivers, fewer organizational issues, and less difficulty taking leave. Above this threshold, all nine heightened stressors were significantly more prevalent. These findings emphasize how appropriate nurse staffing may alleviate stressors associated with workload, teamwork, and job conditions.

### 4.1. Possible Mechanisms Underlying the Association Between PNR and Stress

Higher PNRs have been associated with increased work demands, making it difficult for nurses to provide adequate patient care [36, 37]. Participants in this study reported that they had insufficient time to offer mental health care to patients (“Powerlessness” subscale), and excessive duties prevented them from attending to patients (“Work Demands” subscale). These findings are consistent with prior studies showing that high patient loads reduce nurses' ability to perform essential care tasks and increase perceived work pressure [6, 29, 38].

In addition, when patient loads were high, nurses experienced stress because of primary caregivers failing to execute tasks appropriately and were concerned about colleagues' incompetence affecting patient safety (“Insufficient Support from Coworkers or Caregivers” subscale). These stressors may be exacerbated under high workloads because of reduced peer support and fewer opportunities for senior nurses to mentor junior staff. Prior studies have shown that understaffing is closely linked to weakened team collaboration and diminished mutual assistance among nurses [24, 29]. In addition, heightened workloads may strain interprofessional collaboration, leading to miscommunication or inefficient task delegation [39]. In Taiwan, reliance on family members or privately hired aides to assist with inpatient care further complicates the issue. While nurses often provide basic instructions to these caregivers, the quality and consistency of task execution may vary, potentially undermining patient care. Future research should explore whether structured training programs and caregiver support systems could mitigate these effects.

Beyond these direct effects, higher PNRs may intensify nurses' emotional labor by forcing them to ration care and suppress emotions while managing overwhelming workloads. In high-demand environments, nurses may experience emotional disconnection as a coping mechanism when they were unable to provide the level of care they aspired to deliver. The phrase “greatest good for the greatest number” encapsulated the reality that time constraint and staffing shortages compelled nurses to prioritize tasks pragmatically, leading to emotional strain [38]. Emotional labor has been closely linked to stress, burnout, and turnover intention among nurses [40]. However, because of data limitations, emotional labor was not measured in this study. Future research should employ mediation analysis to refine staffing policies that address both direct and indirect nurse workload effects.

If unaddressed, these stressors may exacerbate nurse burnout and compromise patient care quality, highlighting the need to assess PNR's broader impact on workforce sustainability and healthcare outcomes.

### 4.2. Broader Implications for Nurse Well-Being and Patient Care

While initially affecting nurse well-being, stressors such as excessive workloads, insufficient rest, and poor staffing policies may ultimately compromise patient care if not properly managed. For instance, the inability to take uninterrupted breaks (“Unmet Basic Physiological Needs” subscale) and difficulties in requesting leave (“Difficulty Taking Leave” subscale) may contribute to burnout and fatigue [36, 37, 41], while exposure to psychological abuse, including threats, discrimination, and bullying (“Workplace Violence and Bullying” subscale) may further exacerbate workplace stress [42]. These conditions increase the likelihood of clinical errors and impair decision-making, ultimately affecting care quality.

The protective effects observed at ADPNR < 11 may be attributed to better workload balance, improved teamwork, and enhanced workplace conditions. Lower patient loads allow nurses to devote more time to direct patient care, easing excessive work demands. In addition, manageable workloads may enhance collaboration by enabling senior nurses to mentor junior staff. Reduced staffing-related pressures may also improve scheduling stability, lessen reliance on on-call shifts, and mitigate financial dissatisfaction related to overtime work. These protective effects suggest that lower PNRs could help create a more manageable workload and a stable work environment for nurses. Notably, while this threshold was statistically derived rather than policy based, it may serve as a preliminary reference for minimizing occupational stress. Its applicability may vary across hospital types—tertiary hospitals that manage more complex cases may require even lower PNRs, while community hospitals may have different optimal thresholds. Given the nonlinear association observed in our data, staffing policies should account for not only average PNRs but also promote flexible, shift-specific, and unit-level allocations to better reflect actual workload distribution.

To further support workforce flexibility and mitigate staffing shortages, hospitals could establish staff pools. To build and retain these pools effectively, institutions may offer financial incentives, greater scheduling autonomy, and professional development opportunities to attract and retain qualified nurses [43]. These measures can enhance workforce stability while ensuring timely support for units facing high patient loads or unexpected absences.

Rather than relying on aggregated hospital-wide PNRs, unit-level PNRs may offer a more accurate reflection of nurse workloads. In Japan, the absence of a significant association between PNR and nurse stress may be attributed to reliance on hospital-level reporting, which does not reflect real-time workload variations across different units [25]. Developing predictive models based on unit-level staffing and outcome data could guide more context-specific PNR adjustments.

In addition to PNR regulation, certain settings may warrant priority policy attention. Surgical wards, where patient acuity and workload unpredictability are high, may face heightened stressors related to work demands, insufficient support from coworkers or caregivers, and occupational hazards. Similarly, night shifts and rotating shifts may pose additional risks because of reduced interprofessional support, leading to increased workplace violence and organizational issues. These units may benefit from targeted interventions, such as workload redistribution and enhanced break policies. Aligning work schedules with international guidelines—such as the Japan Nursing Association's recommendation of no more than eight night shifts per month and at least 11 h between shifts to allow for adequate rest and commuting time [44]—could serve as a valuable reference for institutions aiming to enhance nurse well-being and scheduling equity.

Another critical factor contributing to occupational stress is excessive working hours. Long working hours (≥ 55 h per week) were also significantly associated with multiple NOSS subscales, particularly “Work-Family Conflict.” Prolonged working hours contribute to physical exhaustion and increased stress, which may impact both nurse well-being and patient care quality. This finding was consistent with prior research indicating that extended work hours were associated with heightened stress perception and increased risk of burnout among nurses [45]. To mitigate these adverse effects, policies should emphasize work-hour regulations, optimizing nurse staffing to reduce overtime dependency and promoting flexible scheduling systems to better support work-life balance.

### 4.3. Limitations

This study has several limitations that warrant cautious interpretation of the results. First, potential selection bias may exist because of the voluntary participation of hospitals. Hospitals more engaged in research or with stronger staffing management practices may have been more likely to participate, which could limit the representativeness of the sample. In addition, the generalizability of the findings may be constrained by the healthy worker effect [46], as nurses who found it challenging to adapt to their work environment might have exited the profession or transitioned to less demanding roles before the survey, potentially underestimating the true impact of high PNRs on stress levels. In addition, while the overall hospital response rate was relatively high (81.2%), unit-level response rates could not be estimated, limiting a more detailed analysis by department-specific variations in stressors.

The cross-sectional nature of the study also precludes causal inference. While significant associations were identified between ADPNR and stressor subscales, the temporal relationship between staffing levels and stress remains uncertain.

Although the data were collected in 2014, recent reports (2024) from the Taiwan Nurses Union [18] indicate that many hospitals still struggle to meet the 2019 nurse staffing regulations. These findings imply that nurse staffing challenges identified in this study remain relevant to current practice, especially in under-resourced settings.

Moreover, the study did not account for recent technological developments, such as the adoption of electronic medical records, automated scheduling systems, and other digital tools that may influence workload distribution and stress perception. The dataset also predates the COVID-19 pandemic, which has significantly reshaped nursing workloads, safety concerns, and emotional labor demands. These contextual changes may affect the generalizability of the findings to today's healthcare environment. Future studies should incorporate post-pandemic data and explore how digital transformation and systemic reforms influence occupational stress.

Finally, while the ADPNR values in this study ranged from 5 to 20, data for ADPNR levels below 11 were limited (approximately 25% of participants), which restricted the ability to analyze protective effects more fully. Longitudinal research is recommended to better capture temporal dynamics and to examine both direct and indirect mechanisms linking staffing to stress.

## 5. Conclusion

This study highlights the critical associations between PNRs and nurses' occupational stressors, emphasizing the necessity for stricter regulatory oversight and enforcement of ratio standards. Such measures hold the potential to improve working conditions, reduce job-related stress, and enhance patient safety and care quality.

To further mitigate stressors in clinical settings, hospital administrators should consider implementing targeted strategies, such as optimizing staffing levels, revising work schedules, and strengthening support systems. In addition, future research should explore the long-term impacts of PNR regulations on nurse well-being and patient outcomes across diverse healthcare contexts and regions. Such studies would enhance the generalizability of findings and provide robust evidence to inform policy and practice improvements.

## Figures and Tables

**Table 1:** Participants' characteristics (*N* = 996).

Variable	*n* (%)	Mean (SD)
Sex		
Male	18 (1.8)	
Female	950 (95.4)	
Missing	28 (2.8)	
Age (years)		30.6 (6.9)
≦ 25	281 (28.2)	
26–35	492 (49.4)	
≧ 36	207 (20.8)	
Missing	16 (1.6)	
Marital status		
Single/divorced/separated/widowed	656 (65.9)	
Married/cohabitating	337 (33.8)	
Missing	3 (0.3)	
Educational attainment		
Under college	332 (33.3)	
College or above	662 (66.5)	
Missing	2 (0.2)	
Current work tenure (years)		6.8 (6.1)
< 5	514 (51.6)	
5–15	380 (38.2)	
> 15	99 (9.9)	
Missing	3 (0.3)	
Total work tenure (years)		8.2 (6.9)
< 5	431 (43.3)	
5–15	422 (42.4)	
> 15	139 (14.0)	
Missing	4 (0.4)	
Hospital level		
Primary	315 (31.6)	
Secondary	326 (32.7)	
Tertiary	355 (35.6)	
Ownership of the hospital		
Public	380 (38.2)	
Private	616 (61.8)	
Unit type		
Medical ward	623 (62.6)	
Surgical ward	373 (37.4)	
Primary shift over the past 3 months		
Day shift	398 (40.0)	
Evening shift	198 (19.9)	
Night shift	159 (16.0)	
Rotating shift	231 (23.2)	
Missing	10 (1.0)	
Working hours/day		9.5 (1.2)
Working hours/week		47.6 (8.6)
Sleeping hours/day		6.9 (1.3)
Average daily patient-nurse ratio		12.2 (2.6)
Primary hospital		13.1 (3.8)
Secondary hospital		12.6 (1.8)
Tertiary hospital		10.9 (0.9)

Abbreviation: SD, Standard deviation.

**Table 2:** Distribution of the subscales of the nurses' occupational stressor scale (*N* = 996).

Total score of subscales (range: 0–100)	Mean (SD)	25th percentile	Median	75th percentile
1. Work demands	74.1 (16.8)	66.7	77.8	88.9
2. Work-family conflict	66.6 (18.8)	55.6	66.7	77.8
3. Insufficient support from coworkers or caregivers	68.7 (16.3)	55.6	66.7	77.8
4. Workplace violence and bullying	62.6 (24.8)	33.3	66.7	66.7
5. Organizational issues	65.7 (18.3)	55.6	66.7	77.8
6. Occupational hazards	72.7 (16.6)	66.7	66.7	83.3
7. Difficulty taking leave	61.4 (23.9)	50.0	66.7	83.3
8. Powerlessness	65.4 (16.6)	50.0	66.7	66.7
9. Unmet basic physiological needs	69.7 (20.9)	50.0	66.7	83.3

Abbreviation: SD, Standard deviation.

**Table 3:** Associations between the subscales of the nurses' occupational stressor scale and participants' characteristics and work-related factors (*N* = 996).

Variable	Subscales of the nurses' occupational stressor scale								
	Higher work demands	Greater work–family conflict	Lower support from coworkers or caregivers	Increased workplace violence and bullying	More organizational issues	Higher occupational hazards	Greater difficulty taking leave	Increased feelings of powerless-ness	Higher unmet basic physiological needs
Crude odds ratio (95% confidence interval)									
Sex									
Male	1	1	1	1	1	1	1	1	1
Female	1.34 (0.30, 5.88)	1.15 (0.33, 4.02)	1.11 (0.41, 2.98)	1.87 (0.43,8.21)	0.55 (0.22, 1.40)	1.16 (0.44, 3.01)	1.09 (0.39, 3.09)	0.64 (0.24, 1.71)	0.84 (0.33, 2.15)
Age (years)									
≦ 25	1	1	1	1	1	1	1	1	1
26–35	1.38 (0.91, 2.11)	1.32 (0.90, 1.95)	1.59^∗∗^ (1.17, 2.17)	1.25 (0.86, 1.83)	1.34 (0.99, 1.84)	1.20 (0.89, 1.62)	1.86^∗∗∗^ (1.33, 2.60)	0.99 (0.71, 1.40)	1.04 (0.77, 1.40)
≧ 36	0.89 (0.51, 1.55)	1.38 (0.87, 2.18)	0.86 (0.58, 1.27)	1.13 (0.71, 1.80)	0.83 (0.56, 1.22)	1.05 (0.73, 1.52)	1.19 (0.78, 1.81)	0.78 (0.50, 1.19)	0.60^∗∗^ (0.41, 0.87)
Marital status									
Single/divorced/separated/widowed	1	1	1	1	1	1	1	1	1
Married/cohabitating	1.05 (0.73, 1.52)	1.65^∗∗^ (1.19, 2.28)	0.93 (0.71, 1.22)	0.90 (0.64, 1.26)	0.85 (0.65, 1.12)	1.20 (0.92, 1.56)	1.06 (0.80, 1.41)	0.90 (0.66, 1.23)	0.99 (0.76, 1.30)
Educational attainment									
Under college	1	1	1	1	1	1	1	1	1
College or above	1.42 (0.97, 2.11)	1.44^∗^ (1.02, 2.05)	1.19 (0.90, 1.57)	1.04 (0.75, 1.46)	1.12 (0.86, 1.48)	1.40^∗^ (1.07, 1.83)	1.07 (0.80, 1.43)	1.73^∗∗^ (1.25, 2.40)	1.65^∗∗∗^ (1.26, 2.18)
Current work tenure (years)									
< 5	1	1	1	1	1	1	1	1	1
5–15	1.22 (0.85, 1.75)	1.13 (0.81, 1.59)	1.04 (0.79, 1.37)	0.92 (0.65, 1.29)	0.91 (0.69, 1.19)	1.13 (0.87, 1.48)	1.20 (0.90, 1.60)	0.92 (0.67, 1.25)	0.91 (0.69, 1.19)
> 15	0.38^∗^ (0.16, 0.89)	1.15 (0.67, 1.97)	0.93 (0.59, 1.46)	1.11 (0.66, 1.89)	0.66 (0.41, 1.05)	1.21 (0.78, 1.86)	0.66 (0.39, 1.11)	0.89 (0.54, 1.48)	0.59^∗^ (0.37, 0.93)
Total work tenure (years)									
< 5	1	1	1	1	1	1	1	1	1
5–15	1.38 (0.96, 2.01)	1.25 (0.89, 1.77)	1.18 (0.89, 1.56)	1.11 (0.79, 1.55)	0.97 (0.73, 1.29)	1.08 (0.82, 1.41)	1.24 (0.93, 1.67)	0.99 (0.73, 1.35)	0.86 (0.65, 1.12)
> 15	0.54 (0.28, 1.06)	1.22 (0.75, 1.98)	0.83 (0.55, 1.25)	0.96 (0.59, 1.58)	0.65 (0.43, 1.00)	0.93 (0.63, 1.38)	0.82 (0.53, 1.27)	0.82 (0.52, 1.30)	0.51^∗∗^ (0.34, 0.77)
Hospital level									
Primary	1	1	1	1	1	1	1	1	1
Secondary	1.27 (0.81, 1.97)	1.04 (0.70, 1.57)	1.21 (0.88, 1.68)	0.83 (0.57, 1.23)	0.84 (0.61, 1.18)	0.99 (0.73, 1.37)	0.73 (0.52, 1.04)	1.14 (0.79, 1.65)	1.36 (0.98, 1.88)
Tertiary	1.17 (0.76, 1.82)	1.27 (0.86, 1.86)	1.23 (0.89, 1.69)	0.80 (0.54, 1.17)	1.44^∗^ (1.05, 1.97)	1.47^∗^ (1.08, 2.00)	1.03 (0.75, 1.43)	1.30 (0.91, 1.86)	2.22^∗∗∗^ (1.62, 3.04)
Ownership of the hospital									
Public	1	1	1	1	1	1	1	1	1
Private	1.23 (0.86, 1.76)	1.02 (0.74, 1.42)	1.05 (0.80, 1.37)	1.29 (0.94, 1.78)	1.03 (0.79, 1.35)	0.85 (0.66, 1.11)	1.12 (0.85, 1.48)	1.05 (0.78, 1.42)	0.74^∗^ (0.57, 0.97)
Unit type									
Medical ward	1	1	1	1	1	1	1	1	1
Surgical ward	1.66^∗∗^ (1.17, 2.37)	1.25 (0.91, 1.73)	1.37^∗^ (1.05, 1.79)	1.11 (0.82, 1.54)	1.57^∗∗∗^ (1.20, 2.04)	1.57^∗∗∗^ (1.21, 2.04)	1.14 (0.86,1.51)	1.15 (0.85, 1.54)	1.38^∗^ (1.06, 1.79)
Primary shift over the past 3 months									
Day shift	1	1	1	1	1	1	1	1	1
Evening shift	0.71 (0.42, 1.19)	0.77 (0.49, 1.12)	0.85 (0.59, 1.22)	1.01 (0.64, 1.57)	1.27 (0.89, 1.82)	0.95 (0.67, 1.34)	1.10 (0.75, 1.59)	1.27 (0.86, 1.88)	1.10 (0.78, 1.55)
Night shift	0.48^∗^ (0.26, 0.90)	0.71 (0.42, 1.17)	0.95 (0.65, 1.39)	0.64 (0.37, 1.10)	1.07 (0.72, 1.58)	0.90 (0.62, 1.31)	1.06 (0.71, 1.59)	0.84 (0.53, 1.33)	0.76 (0.52, 1.11)
Rotating shift	1.35 (0.88, 2.05)	1.27 (0.86, 1.88)	1.09 (0.78, 1.53)	1.80^∗∗^ (1.22, 2.64)	1.60^∗∗^ (1.14, 2.24)	1.02 (0.74, 1.42)	1.11 (0.79, 1.60)	1.36 (0.94, 1.97)	0.88 (0.63, 1.22)
Working hours/week									
< 55	1	1	1	1	1	1	1	1	1
≧ 55	2.15^∗∗∗^ (1.43, 3.24)	2.94^∗∗∗^ (2.03, 4.25)	1.60^∗∗^ (1.14, 2.24)	1.37 (0.92, 2.04)	2.32^∗∗∗^ (1.66, 3.25)	1.91^∗∗∗^ (1.37, 2.67)	2.06^∗∗∗^ (1.46, 2.90)	2.65^∗∗∗^ (1.87, 3.76)	2.23^∗∗∗^ (1.59, 3.13)
Sleeping hours/day									
≧ 6	1	1	1	1	1	1	1	1	1
< 6	1.39 (0.8, 2.20)	1.94^∗∗^ (1.30, 2.89)	1.83^∗∗∗^ (1.28, 2.60)	1.77^∗∗^ (1.18, 2.65)	1.68^∗∗^ (1.18, 2.40)	1.49^∗^ (1.05, 2.12)	1.33 (0.92, 1.93)	1.59^∗^ (1.08, 2.33)	1.33 (0.94, 1.90)
Average daily patient-nurse ratio	1.14^∗∗∗^ (1.07, 1.21)	1.09^∗∗^ (1.03, 1.15)	1.09^∗∗∗^ (1.04, 1.14)	1.10^∗∗^ (1.04, 1.16)	1.08^∗∗^ (1.03, 1.13)	1.06^∗^ (1.01, 1.11)	1.13^∗∗∗^ (1.08, 1.19)	1.09^∗∗^ (1.03, 1.15)	1.08^∗∗^ (1.03, 1.14)

*Note:* The subscales of the Nurses' Occupational Stressor Scale were defined based on the 75th percentile of each respective subscale, as shown in Table 2.

^∗^ *p* < 0.05.

^∗∗^ *p* < 0.01.

^∗∗∗^ *p* < 0.001.

**Table 4:** Associations between the subscales of the nurses' occupational stressor scale and standardized average daily patient-nurse ratio (*N* = 996).

Variable	Subscales of the nurses' occupational stressor scale								
	Higher work demands	Greater work–family conflict	Lower support from coworkers or caregivers	Increased workplace violence and bullying	More organizational issues	Higher occupational hazards	Greater difficulty taking leave	Increased feelings of powerless-ness	Higher unmet basic physiological needs^a^
Adjusted odds ratio (95% confidence interval)									
Age (years)	—	—		—	—	—		—	—
≦ 25			1				1		
26–35			1.60^∗∗^ (1.17, 2.19)				1.82^∗∗∗^ (1.30, 2.57)		
≧ 36			0.83 (0.56, 1.24)				1.14 (0.74, 1.75)		
Marital status	—	—	—	—	—	—	—	—	—
Single/divorced/separated/widowed		1							
Married/cohabitating		1.77^∗∗^ (1.26, 2.48)							
Educational attainment	—		—	—	—		—		
Under college		1				1		1	1
College or above		1.74^∗∗^ (1.20, 2.54)				1.24 (0.91, 1.69)		1.95^∗∗∗^ (1.38, 2.74)	1.22 (0.88, 1.70)
Current work tenure (years)		—	—	—	—	—	—	—	—
< 5	1								
5–15	1.19 (0.82, 1.74)								
> 15	0.40^∗^ (0.17, 0.96)								
Total work tenure (years)	—	—	—	—	—	—	—	—	
< 5									1
5–15									0.97 (0.73, 1.30)
> 15									0.53^∗∗^ (0.34, 0.82)
Hospital level	—	—	—	—			—	—	
Primary					1	1			1
Secondary					0.97 (0.68, 1.40)	0.97 (0.69, 1.35)			1.59^∗^ (1.11, 2.28)
Tertiary					2.31^∗∗∗^ (1.55, 3.45)	1.64^∗∗^ (1.16, 2.32)			3.92^∗∗∗^ (2.39, 6.42)
Ownership of the hospital	—	—	—	—	—	—	—	—	
Public									1
Private									1.41 (0.97, 2.05)
Unit type		—		—			—	—	
Medical ward	1		1		1	1			1
Surgical ward	1.96^∗∗∗^ (1.35, 2.85)		1.48^∗∗^ (1.12, 1.94)		1.55^∗∗^ (1.17, 2.06)	1.54^∗∗^ (1.17, 2.01)			1.19 (0.90, 1.58)
Primary shift over the past 3 months		—	—			—	—	—	—
Day shift	1			1	1				
Evening shift	0.72 (0.43, 1.22)			0.99 (0.64, 1.57)	1.34 (0.92, 1.94)				
Night shift	0.51^∗^ (0.27, 0.97)			0.60 (0.35, 1.05)	1.12 (0.74, 1.68)				
Rotating shift	1.31 (0.84, 2.03)			1.68^∗∗^ (1.14, 2.49)	2.06^∗∗∗^ (1.41, 3.00)				
Working hours/week									
< 55	1	1	1		1	1	1	1	1
≧ 55	1.86^∗∗^ (1.21, 2.87)	2.78^∗∗∗^ (1.90, 4.07)	1.54^∗^ (1.08, 2.19)		2.24^∗∗∗^ (1.57, 3.19)	1.91^∗∗∗^ (1.35, 2.69)	1.89^∗∗∗^ (1.32, 2.70)	2.49^∗∗∗^ (1.74, 3.57)	2.14^∗∗∗^ (1.50, 3.05)
Sleeping hours/day	—						—		—
≧ 6		1	1	1	1	1		1	
< 6		1.92^∗∗^ (1.26, 2.91)	1.63^∗∗^ (1.13, 2.35)	1.65^∗^ (1.08, 2.51)	1.41 (0.97, 2.06)	1.38 (0.96, 1.98)		1.50^∗^ (1.01, 2.23)	
Average daily patient-nurse ratio	1.13^∗∗∗^ (1.06, 1.20)	1.07^∗^ (1.01, 1.13)	1.07^∗^ (1.02, 1.13)	1.09^∗∗^ (1.03, 1.15)	1.12^∗∗∗^ (1.06, 1.19)	1.08^∗∗^ (1.02, 1.14)	1.11^∗∗∗^ (1.05, 1.17)	1.08^∗∗^ (1.02, 1.14)	1.18^∗∗∗^ (1.10, 1.25)

*Note:* The subscales of the Nurses' Occupational Stressor Scale were defined based on the 75th percentile of each respective subscale, as shown in Table 2.

^a^Because of high correlations among age, total work tenure, and current work tenure (*r* = 0.90 between age and total tenure; *r* = 0.78 between total and current tenure), only one was included in each adjusted model to avoid multicollinearity.

^∗^ *p* < 0.05.

^∗∗^ *p* < 0.01.

^∗∗∗^ *p* < 0.001.

## Data Availability

The data that support the findings of this study are available from the corresponding author upon reasonable request.
